# Application of headless cannulated compression screws for treatment of Delbet-Colonna II and III femoral neck fractures in children

**DOI:** 10.3389/fped.2025.1660855

**Published:** 2025-08-22

**Authors:** Yikun Jiang, Yanbing Wang, Chuangang Peng, Baoming Yuan, Dankai Wu

**Affiliations:** Department of Orthopedics, The Second Hospital of Jilin University, Changchun, China

**Keywords:** closed reduction, femoral neck fracture, headless cannulated compression screw, internal fixation, Delbet-Colonna II and III, children patient

## Abstract

**Purpose:**

Femoral neck fractures are clinically rare and are associated with a high risk of complications in children. Traditional internal fixation implants such as Kirschner wires and partial-thread cannulated screws (PTCS) have complications such as screw withdrawal and internal fixation failure. To address this problem, in this study we investigated the effectiveness of headless cannulated compression screws (HCCS) in the treatment of femoral neck fractures in children patients.

**Methods:**

Children diagnosed with Delbet-Colonna II or III femoral neck fracture treated by closed reduction and percutaneous fixation with HCCS were retrospectively reviewed. The extent of fracture reduction and postoperative hip function were assessed according to the Haidukewych standard and with the Harris score, respectively. Postoperative complications were recorded.

**Results:**

According to the inclusion criteria and exclusion criteria in this retrospective study, A total of 12 patients (8 males and 4 females) aged 3–14 years (average age: 8.3 years) were reviewed. The mean blood loss from surgery was 34.58 ± 9.40 ml and mean operation time was 102.50 ± 32.72 min. Overall, fracture reduction was achieved in most cases, with 7 that were excellent (58.33%) and 5 that were good (41.67%) according to the Haidukewych standard. The average follow-up period was 24.67 months. Radiographic analysis revealed an average time for fracture healing of 8.58 ± 3.87 weeks. Harris score was 88.67 ± 2.61 at 3 months after surgery, and increased to 92.25 ± 1.91 at the 6-month follow-up; excellent outcomes were achieved at the last follow-up evaluation (95.17 ± 1.95). No surgery-related complications were reported during the follow-up period.

**Conclusions:**

We recommend closed reduction and internal fixation with HCCS as a feasible alternative for the treatment of Delbet-Colonna II and III femoral neck fractures in children.

## Introduction

Femoral neck fractures in children are clinically rare but have a high risk of complications including avascular necrosis, coxa valga, nonunion, deformity, and premature physeal closure (1–3). For optimal clinical outcomes in children patients, fixation implants must be carefully chosen to achieve stable fixation of the femoral neck with minimal damage to the bone cortex and physis.

Delbet-Colonna types II and III are the femoral neck fractures in children most commonly seen in clinical practice (4). In general, they are treated by internal fixation using Kirschner wires or partially thread cannulated screws (PTCS). However, the former has relatively low mechanical performance and require additional immobilization with a spica cast, which is a burden in the postoperative care of children patients (5, 6). On the other hand, PTCS may not function normally in patients with lateral cortex injuries of the femoral neck or who experience bone absorption after surgery, leading to screw withdrawal and failure of internal fixation (7, 8). There is therefore a need for an appropriate substitute fixation implant with superior biomechanical performance.

Headless cannulated compression screws (HCCS) have demonstrated superior biomechanical stability and good clinical outcome in the treatment of adult femoral neck fractures (9). The full-thread headless design has greater holding force and pullout and shear strengths and minimizes cortex damage (10), in contrast to Kirschner wires and PTCS. However, the application of HCCS to femoral neck fractures in children has not been previously reported.

To this end, in the present study we investigated the effectiveness of HCCS as substitute for traditional fixation implants in the treatment of femoral neck fracture in children.

## Materials and methods

### Ethics approval and consent to participate

This study was conducted in accordance with the principles outlined in the Declaration of Helsinki and was approved by the Ethics Committee of the Second Hospital of Jilin University (2020-016). Written, informed consent to participate was obtained from all patients involved in the study. Patient data were kept anonymous to ensure confidentiality and privacy.

### Inclusion and exclusion criteria

The inclusion criteria were as follows: 1. child patients <15 years old; 2. clinically diagnosed with acute femoral neck fracture; 3. available for follow-up for >6 months after surgery; and 4. no abnormal gait pattern or movement disorder before the current injury. The exclusion criteria were as follows: 1. diagnosed with congenital hip diseases or severe metabolic diseases or with pathologic, old, or open fractures; 2. underwent open reduction; 3. underwent internal fixation with traditional PTCS or Kirschner wires; and 4. underwent closed reduction and spica cast immobilization.

### Study design and participants

**Figure 1:**
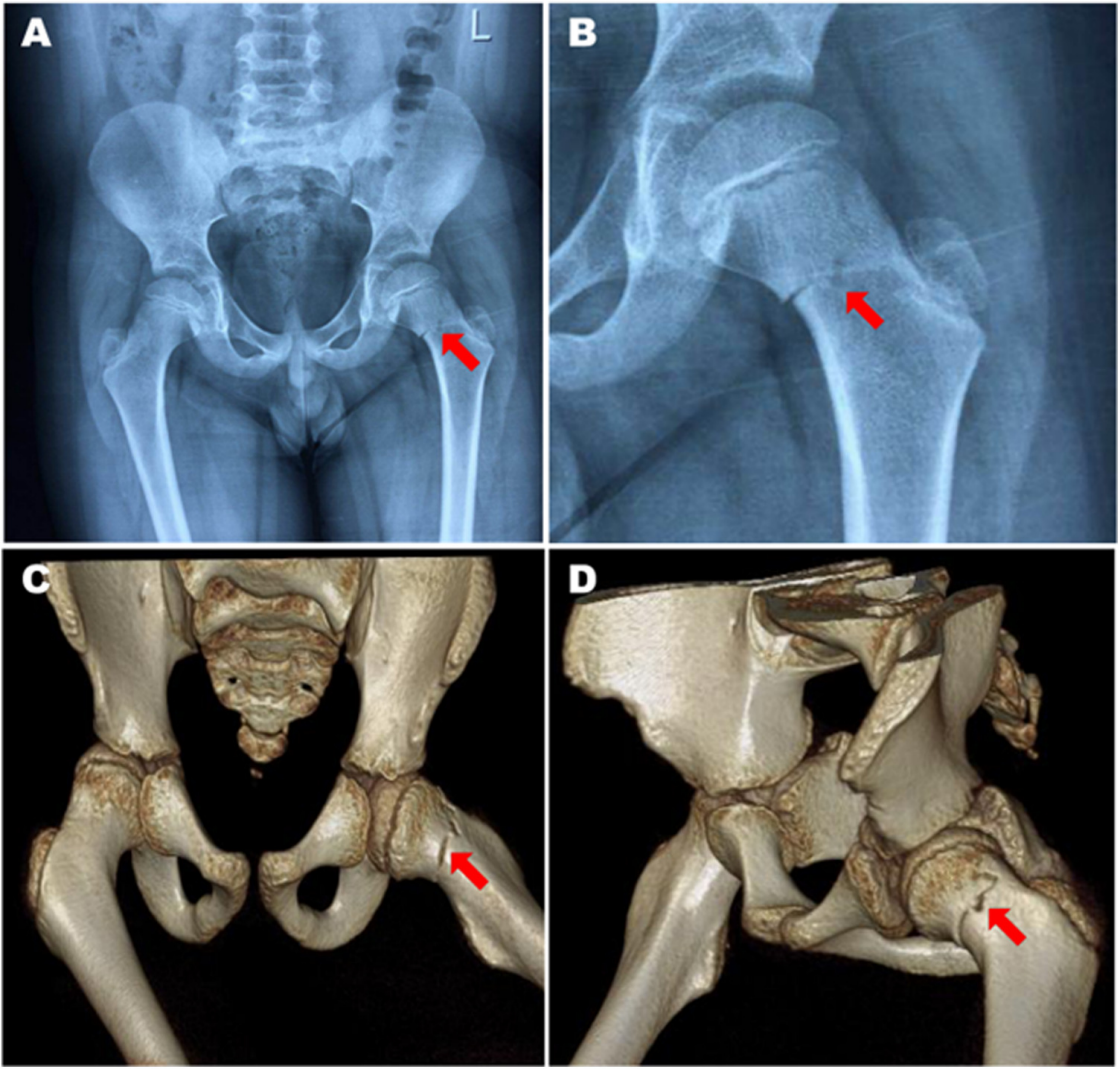
An 8-year-old male patient with Delbet-Colonna type II femoral neck fracture. **(A,B)** Preoperative x-rays showed minor displacement of the fracture with an indistinct fracture line. **(C,D)** The femoral neck fracture was confirmed by 3-dimensional computed tomography.

In this retrospective study, we collected and analyzed the clinical data of 12 children patients treated for femoral neck fracture between May 2014 and February 2019 at our orthopedic center. The mean age of patients was 8.33 years (3–14), with 8 males and 4 females. The main causes of high-impact injury were traffic accident, falling from a height, and daily activities. All patients were evaluated by x-ray radiography (Winscope Plessart EX8, Canon.Inc) of the hip (Figures 1A,B) for diagnosis and Delbet-Colonna classification. Computed tomography scans (Ingenuity, Philips Inc.) were performed along with 3-dimensional reconstruction when the fracture was unclear or difficult to classify based on radiographs (Figures 1C,D). There were 5 and 7 cases of Delbet-Colonna type II and III, respectively. The characteristics of the study population are summarized in Table 1.

**Table 1:** Detailed information regarding patient characteristics and fractures.

Index	Value
Male/female	8/4
Age	8.33 years (3–14)
Right/left	6/6
Cause of injury	
Motor vehicle crash	2 (16.7%)
Fall during activity	6 (50.0%)
Fall from a height	4 (33.3%)
Isolated femoral neck fracture	9 (75.0%)
Multiple fractures	3 (25.0%)
Fracture type	
Delbet-Colonna type II	5 (41.7%)
Delbet-Colonna type III	7 (58.3%)
Fracture displacement	
Minor	7 (58.3%)
Obvious	5 (41.7%)
Admission after injury	
Within 24 h	9 (75.0%)
2–7 Days	2 (16.7%)
Over 7 days	1 (8.3%)
Fixation after injury	
12–24 h	5 (41.7%)
Over 24 h	7 (58.3%)

### Preoperative preparation

All patients received temporary skin traction of the injured limb with approximately 1/8 of their body weight immediately after admission until the surgery was performed. The surgery was performed as soon as the general condition of the patient permitted.

### Surgical techniques

**Figure 2:**
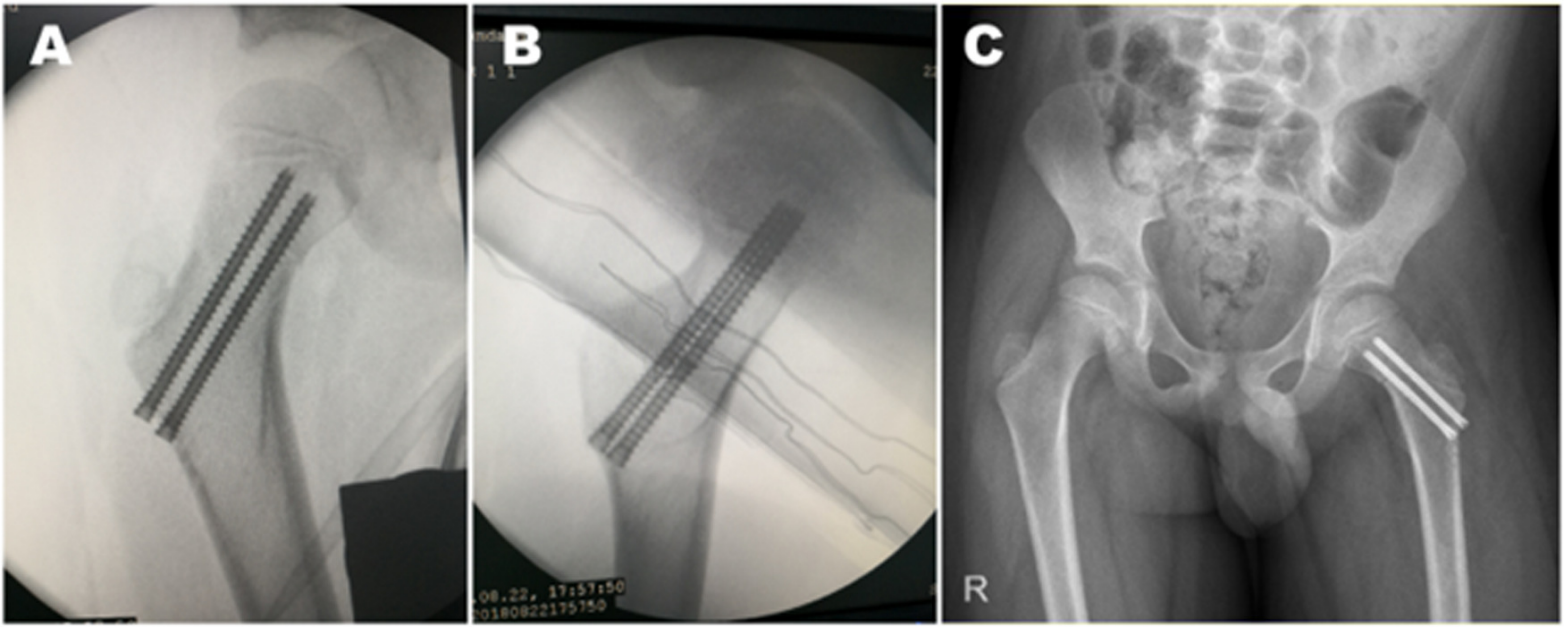
X-ray radiography during the surgery. **(A–C)** Two HCCSs were inserted after closed reduction of the fracture, and the extent of fracture reduction was evaluated based on anteroposterior and lateral x-ray radiographs obtained during the surgery **(A,B)** and anteroposterior radiographs obtained on the day after surgery **(C).**

All operations were performed under general anesthesia. The patient was placed on the traction table in the supine position. The injured limb was placed in the Von-Rosen position. Closed reduction of the femoral neck fracture was performed with C-arm x-ray (SXT-1000A, Canon.Inc) imaging. Following fracture reduction, percutaneous fixation with HCCS was performed according to standard procedures. A Kirschner wire was drilled along the direction of the femoral neck at the lesser trochanter level from the lateral cortex under the greater trochanter. The second Kirschner wire was drilled along the center of the femoral neck 0.5–1 cm away from the epiphyseal plate. For children with a larger femoral neck, an additional Kirschner wire was drilled parallel to the second one for enhanced stability. The position of the Kirschner wires was confirmed by radiography; the wires served as a guide for the inserted HCCS. The screw incision was closed after radiographic confirmation of stable fracture fixation (Figures 2A,B). The Haidukewych standard (11) was used to evaluate the extent of reduction of the femoral neck fracture, which was graded based on the degree of residual angulation and amount of displacement as excellent (displacement <2 mm and angulation <5° in any plane), good (displacement of 2–5 mm and/or angulation of 5°–10°), fair (displacement >5 mm up to 10 mm and/or angulation >10° up to 20°), or poor (displacement >10 mm and/or angulation >20°).

### Postoperative treatment and follow-up

Anteroposterior radiographic examination of the hip was performed 1 day after the surgery (Figure 2C). None of the patients underwent further spica cast immobilization. The patients began isometric contraction training of lower limb muscles on postoperative day 2 and active/passive functional exercises of the hip at day 3. Only ambulated with crutches and toe-touch weight bearing was allowed until the follow-up examination showed that the fracture was consolidated. The patients engaged in general rehabilitation exercises under the guidance of the surgeons.

Nonphysiologic closure of the femoral epiphysis or bridge formation during follow-up was diagnosed as premature physeal closure (12). In patients whose fracture began healing within 6 months, the internal fixation was removed 1 year after surgery. Harris score (13) was used to evaluate hip function after surgery (excellent, >90; good, 80–89; fair, 70–79; and bad, 69).

### Statistical analysis

Results in this study are descriptiveare and are presented in the form of as mean ± standard deviation. Statistical analyses were performed using SPSS v22.0 software (SPSS Inc., Chicago, IL, USA).

## Results

### Operation and short-term complications evaluation

The operation was completed successfully in all patients. The mean volume of blood lost during surgery was 34.58 ± 9.40 ml and the mean operation time was 102.50 ± 32.72 min. Femoral neck fracture reduction was achieved in all patients, with 7 cases that were excellent (58.33%) and 5 that were good (41.67%) according to the Haidukewych standard. The screw incision of all patients healed within 14 days and no signs of wound infection, skin necrosis, or other surgical complications were observed.

### Clinical evaluation and final follow-up

**Figure 3:**
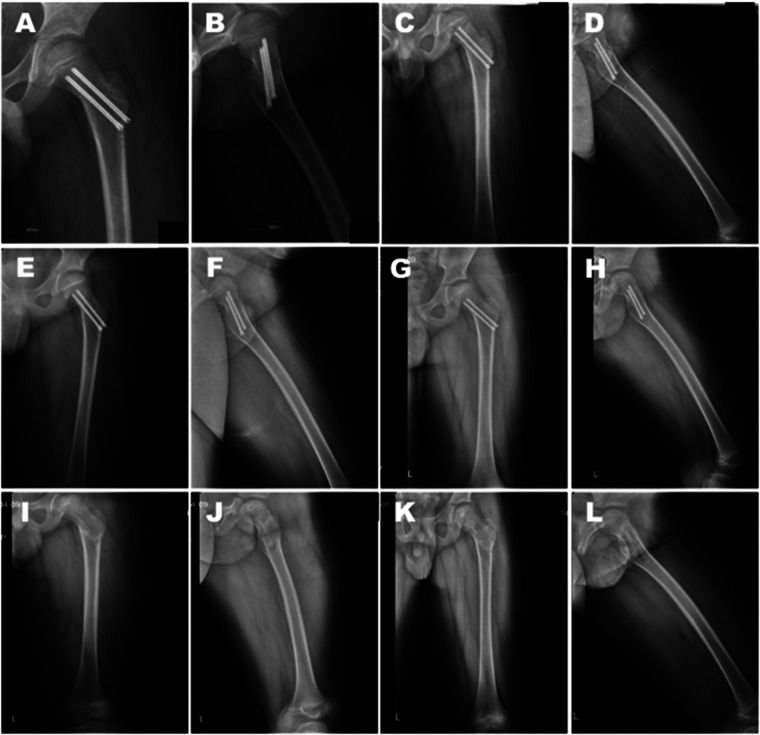
Postoperative anteroposterior and lateral x-ray radiographs. **(A–H)** The patient was examined at 1 day **(A,B)**, 1.5 months **(C,D)**, 4 months **(E,F)**, and 10 months **(G,H)** after surgery, as well as 1 day **(I,J)** and 1 month **(K,L)** after implant removal.

**Figure 4:**
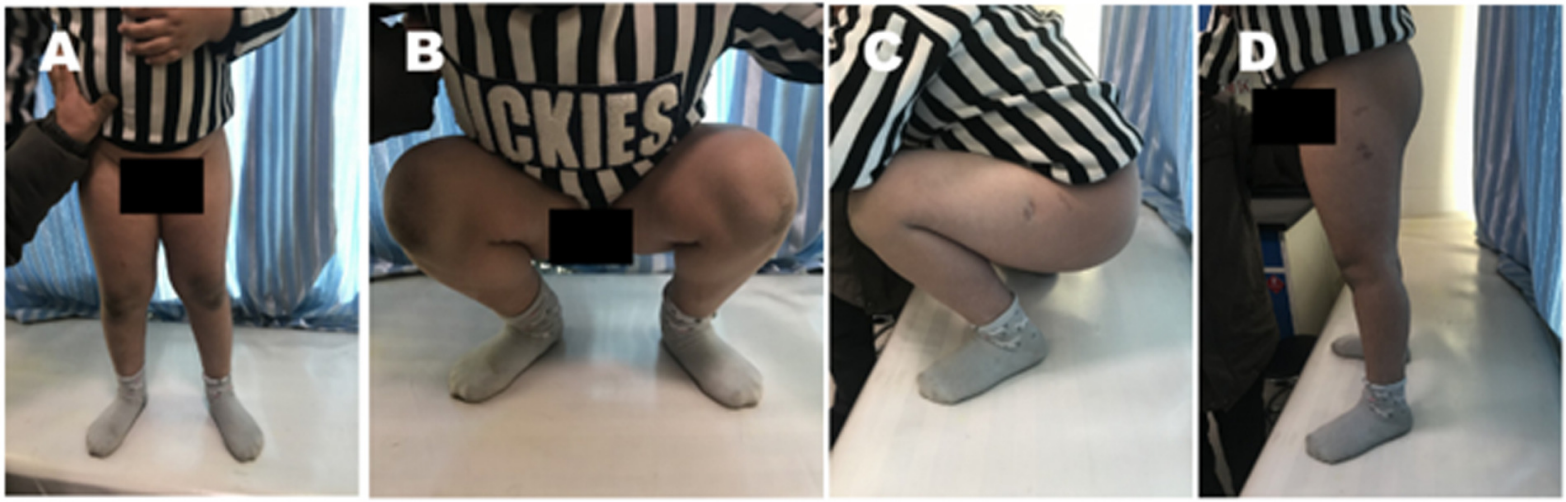
Functional recovery of a patient post surgery. **(A–D)** Anteroposterior **(A,B)** and lateral **(C,D)** images of the patient in erect and squatting positions 4 months after surgery. The patient was followed up for 18 months. No postoperative complications were reported. The last Harris score was 93.

The average follow-up period was 24.67 (7–45) months; 1 case was followed up for <12 months. Anteroposterior and lateral x-ray radiographs (Figure 3) as well as functional images (Figure 4) were obtained. Radiographic examination revealed that the average time for fracture healing was 8.58 ± 3.87 weeks. No procedure-related complications occurred during the follow-up period. Implants were removed after an average 12.17 months. Harris score was 88.67 ± 2.61 at 3 months post surgery and had increased to 92.25 ± 1.91 at the 6-month follow-up; excellent outcomes were recorded at the last follow-up evaluation (95.17 ± 1.95). No surgery-related complications occurred during the follow-up period (Table 2).

**Table 2:** Postoperative follow-up outcomes regarding operation and functional recovery.

Index	Value
Average operation time (min)	102.50 ± 32.72
Average blood loss (ml)	34.58 ± 9.40
Extent of fracture reduction	
Excellent	7 (58.3%)
Good	5 (41.7%)
Fair	0
Poor	0
Radiographic healing time (weeks)	8.58 ± 3.87
Follow-up time (months)	24.67 ± 13.21
Average Harris score	
After 3 months	88.67 ± 2.61
After 6 months	92.25 ± 1.91
At last follow-up	95.17 ± 1.95

## Discussion

The choice of fixation device in treating femoral neck fractures in children depends on multiple factors. In general, Kirschner wires, PTCS, and locking compression hip plates (LCHPs) are used. Kirschner wires are traditionally recommended to prevent potential damage to vasculature and the epiphyseal plate (12); however, their use is associated with a high risk of complications. In Delbet-Colonna type II and III children patients treated using Kirschner wires, a complication rate of 25% was reported that included osteonecrosis, premature physeal closure, and coxa vara (5). Children patients require additional immobilization with a spica cast for 4–6 weeks after internal fixation with Kirschner wires, which increases the complexity and difficulty of postoperative medical care (6). Additionally, in children patients a broken pin can occasionally penetrate the femoral neck and even the acetabulum during daily activities, resulting in unexpected complications (14, 15). In our study, no such complications were observed; owing to the stable fixation achieved by HCCS, all patients were able to begin rehabilitation training on the second day after surgery. The complications of a broken wire and penetration were also avoided.

PTCS compress fracture ends by squeezing the screw caps and outer cortex of the femoral neck (16). However, in patients with lateral cortex injuries or post-surgery bone absorption, screw caps can separate from the femoral cortex, leading to screw withdrawal, failure of internal fixation, and serious complications such as nonunion and limb shortening (9, 17). An average shortening of 1.8 cm and average external rotation of the femoral neck of 44° were reported in young adult patients treated with PTCS (7). Thus, there is still a need for internal fixation implants with greater stability.

LCHPs have better mechanical performance than PTCS and Kirschner wires as they transfer nearly all mechanical force from the femoral neck to the plate (18). However, the 3 screws of LCHPs in the femoral neck apply no compression force to the fracture (19). Moreover, use of LCHPs for the treatment of femoral neck fractures in children requires open reduction surgery, which can cause extensive damage to the bone cortex, surrounding tissues, and vasculature, thereby delaying bone healing and hospital stay and leaving large surgical scars in children patients (20).

HCCS are designed in a cone shape, with the diameter of the tail larger than that of the tip, which perfectly matches the tapered anatomical structure of the femoral neck in children (21). When screwed into bone, the screw tip enters more rapidly than the tail, causing compression between fractures. Moreover, the full-thread design increases the contact area between screw and bone, thus increasing holding force, pullout strength, and shear strength. HCCS showed better performance in the maximum load to failure test and had greater biomechanical stability than PTCS when used to treat vertical femoral neck fracture (9). Applying non-sliding, full-thread constructs can achieve a high union rate with minimal shortening of the femoral neck compared to partially threaded devices by reducing the rate of fixation failure (22). Moreover, the headless design of HCCS minimizes disturbance to surrounding tissues and avoids potential damage to the blood supply of the femoral head (23). In our study, all patients underwent internal fixation with HCCS. The follow-up examinations showed no evidence of screw withdrawal or femoral neck shortening; all 12 cases achieved complete fracture union during the follow-up period. The average bone reunion time was 8.58 ± 3.87 weeks, which is significantly shorter than that reported with open reduction (10.87 ± 1.59 weeks) (24) and for femoral neck fractures in children treated with Kirschner wires (10.0 ± 1.33 weeks) and PTCS (10.4 ± 1.28 weeks) (25). These results provide evidence for the stability and durability of HCCS as an internal fixation device in children Delbet-Colonna II and III femoral neck fractures.

The present study had limitations. Firstly, it had a retrospective design and there was no control group. Given the low rate of femoral neck fractures in children, the number of patients was small, with only Delbet-Colonna type II and III cases. Secondly, since this study is a retrospective study, the various information collected may lead to errors and cause deviations in the results. Thirdly, as the follow-up period in some cases was relatively short, the frequency of long-term complications such as premature physeal closure and coxa vara remains to be determined. Fouthly, we did not set up a control group for other surgical methods to compare the advantages of this procedure.

## Conclusion

In this study we examined the intra- and postoperative effectiveness of HCCS fixation in the treatment of Delbet-Colonna II and III femoral neck fractures in children. HCCS fixation produced excellent clinical outcomes including fracture reduction and early recovery with no signs of complications, demonstrating that it is a feasible option for the treatment of femoral neck fractures in children. However, this study has the limitations of a small number of cases and being a retrospective study. In future research, more patients need to be included to enhance the feasibility of the surgical method

## Data Availability

The datasets presented in this study can be found in online repositories. The names of the repository/repositories and accession number(s) can be found in the article/supplementary material.
